# Expression and Intracellular Localization of Paraoxonase 2 in Different Types of Malignancies

**Published:** 2018

**Authors:** M. I. Shakhparonov, N. V. Antipova, V. O. Shender, P. V. Shnaider, G. P. Arapidi, N. B. Pestov, M. S. Pavlyukov

**Affiliations:** Shemyakin–Ovchinnikov Institute of Bioorganic Chemistry, Miklukho-Maklaya Str., 16/10, Moscow, 117997, Russia

**Keywords:** paraoxonase, cancer, apoptosis, glioblastoma, protein-protein interactions

## Abstract

PON2 belongs to the paraoxonase protein family that consists of lactone hydrolyzing enzymes with different substrate specificities. Unlike other members of the family, PON2 exhibits substantial antioxidant activity, is localized predominantly inside the cell, and is ubiquitously expressed in all human tissues. Previously, it was proffered that defense against pathogens, such as *Pseudomonas aeruginosa*, is the main function of paraoxonases. However, recent findings have highlighted the important role played by PON2 in protection against oxidative stress, inhibition of apoptosis, and progression of various types of malignancies. In the current study, we performed a bioinformatic analysis of RNA and DNA sequencing data extracted from tumor samples taken from more than 10,000 patients with 31 different types of cancer and determined expression levels and mutations in the *PON2 *gene. Next, we investigated the intracellular localization of PON2 in multiple cancer cell lines and identified the proteins interacting with PON2 using the LC-MS/MS technique. Our data indicate that a high PON2 expression level correlates with a worse prognosis for patients with multiple types of solid tumors and suggest that PON2, when localized on the nuclear envelope and endoplasmic reticulum, may protect cancer cells against unfavorable environmental conditions and chemotherapy.

## INTRODUCTION

The paraoxonase family comprises three enzymes: PON1, PON2, and PON3. A phylogenetic analysis of these proteins seemed to demonstrate that PON2 is the most ancient member of this family, which later gave rise to PON1 and PON3 during evolution [1]. All these enzymes exhibit a pronounced lactonase activity but differ in terms of substrate specificity. Furthermore, paraoxonases have different expression profiles. Hence, PON1 and PON3 are synthesized by the liver; in blood plasma, the proteins are associated with high-density lipoproteins. Unlike those, PON2 is ubiquitously expressed in all human tissues and is localized mostly within the cell. Interestingly, the main function of PON2 in some cell types is related to the antioxidant activity of this enzyme [2]. Thus, PON2 has been shown to significantly reduce the production of superoxide ions as it interacts with complex I and III of the electron transport chain on the inner mitochondrial membrane and also participates in peroxidation of lipids in the plasma membrane [3]. The antioxidant activity of PON2 is independent of the lactonase activity of this enzyme [4].

Detailed studies focused on the structure of PON2 have demonstrated that this protein weighs ~40 kDa, carries two glycosylation sites, a short intracellular domain (1–5 a.a.), a transmembrane domain consisting of a single α-helix (6–24 a.a), as well as a hydrophobic domain (67–81 a.a.) and a enzymatic domain (168–246 a.a.) residing on the outer side of the plasma membrane. Due to its transmembrane domain, PON2 is incorporated into the lipid bilayer during translation and is distributed between the endoplasmic reticulum, the perinuclear region, mitochondria, and the plasma membrane. However, data on the predominant localization of PON2 inside the cell is rather controversial [3, 5, 6, 7].

There has recently been a keen interest in paraoxonase 2, as this protein was found to be associated with malignancy progression. Over the past year, many laboratories have described the important role played by PON2 in tumor cells. Hence, they have demonstrated that PON2 contributes to the progression and metastasizing of pancreatic cancer by stimulating glucose uptake [8], accelerates the proliferation of and resistance to oxidative stress in bladder cancer [9], protects glioblastoma cells against apoptosis [10], and reduces the sensitivity of oral cancer cells to radiation therapy [11]. However, the exact role played by PON2 in other cancer types is yet to be elucidated.

## MATERIALS AND METHODS

**Cell Culture**

Cells were grown in air enriched with 5% (v/v) CO_2_ at 37oC in Dulbecco’s modified Eagle’s medium (DMEM) supplemented with 10% (v/v) fetal bovine serum (FBS) and a 2mM L-glutamine and penicillin (100 units/ml) streptomycin (100μg/ml) mixture. The cells were transfected with the Lipofectamine LTX reagent (Thermo Fisher Scientific; USA) according to the manufacturer’s protocol.

**Immunofluorescence microscopy**

The cells were washed 3 times with phosphate buffered saline (PBS) and fixed with 4% PFA in PBS for 15 min at room temperature. Next, the cells were washed 2 times with PBS and permeabilized with 0.2% Triton-X100 in PBS for 15 minutes. After permeabilization, the cells were blocked for 5 min with 1% BSA in PBST (0.1% Tween 20 in PBS). Next, they were incubated with primary antibodies against PON2 (1:200 dilution; HPA029193, Sigma) or against CRM1 (1:200 dilution; NB100-79802, Novus Biologicals) in PBST for 1 hour. Then, they were washed 5 times with PBST and incubated for an additional hour with secondary antibodies conjugated with Alexa Fluor 555 (dilution 1:500; A32732, Thermo Fisher Scientific) in PBST. Finally, the cells were washed 6 times with PBST to remove unbounded secondary antibodies and stained with DAPI (4’,6-diamidino-2-phenylindole). After 10 min of incubation, the cells were analyzed under a fluorescent microscope.

**Plasmid Construction**

The DNA fragment encoding PON2 was amplified from the previously obtained one by the PCR technique using the primer pair BglII-PON2 (AAA AAG ATC TAT GGG GCG GCT GGT GGC TGT G) and PON2-SalI (AAA AGT CGA CAG TTC ACA ATA CAA GGC TCT GTG GTA) and cloned into the BglIII/SalI sites of the pTurboGFP-N or pTagRFP-C plasmid (Evrogen) to generate the pTurboGFP-N-PON2 and pTagRFP-C-PON2 plasmids, respectively. The DNA fragment encoding 1-27 a.a. of PON2 was amplified from the previously obtained one by the PCR technique using the primer pair BglII-PON2 and PON2_rev2 (TAT TGT CGA CAG TCG ATT TCT GAG TGC CA) and cloned into the BglIII/SalI sites of the pTurboGFP-N plasmid to generate the pTurboGFP-N-PON2-1 plasmid. The DNA fragment encoding 1-83 a.a. of PON2 was amplified from the previously obtained one by the PCR technique using the primer pair BglII-PON2 and PON2_rev3 (AAT TGT CGA CCC TCC AGG CTT ATC T) and cloned into the BglIII/SalI sites of the pTurboGFP-N plasmid to generate the pTurboGFP-N-PON2-2 plasmid. The DNA fragment encoding 1-168 a.a. of PON2 was amplified from the previously obtained one by the PCR technique using the primer pair BglII-PON2 and PON2_rev4 (ATT TGT CGA CAT GTC ATT CAC ACT TGG A) and cloned into the BglIII/SalI sites of the pTurboGFP-N plasmid to generate the pTurboGFP-N-PON2-3 plasmid. For overexpression of full length PON2 fused to Halo-tag, we amplified the Halo-tag sequence from the pFC20K HaloTag T7 SP6 Flexi plasmid (Promega) by the PCR technique using the primer pair SalI-Halo (AGG AGT CGA CTG AGG ATC TGT ACT TTC A) and Halo-NotI (GAG GGC GGC CGC TTA ACC GGA AAT CTC CAG AGT A) and cloned into the SalI/NotI sites of the pTurboGFP-N-PON2 plasmid. Thus, we had replaced the GFP coding sequence with the Halo-tag coding sequence. The resulting plasmid was named pTurboHALO-N-PON2. In all cases, the absence of unwanted mutations in the inserts and vector-insert boundaries was verified by sequencing.

**Purification of PON2 interacting proteins**

U87MG cells were grown on a T75 flask and transfected with the pTurboHALO-N-PON2 plasmid. Forty-eight hours after transfection, the cells were dissociated by a Trypsin-Versene solution and washed twice with ice-cold PBS. Next, the cells were lysed in mammalian cell lysis buffer (50 mM Tris-HCl, 150 mM NaCl, 1% Triton X100, 0.1% sodium deoxycholate, 1 mM PMSF, pH 7.5). Lysate was centrifuged for 15 min, 20,000 g at 4°C. After this, the PON2-interacting proteins were purified with Magne HaloTagBeads (Promega), according to the manufacturer’s protocol.

**Trypsin digestion**

The proteins were eluted from magnetic beads through 30-min incubation with a buffer containing 8M Urea, 2M Thiourea, and 10 mM Tris (pH 8). Then, protein disulfide bonds were reduced with 5 mm DTT at RT for 30 min and, afterwards, alkylated with 10 mm iodoacetamide at room temperature for 20 min in the dark. Next, the samples were diluted (1:4) with 50 mM ammonium bicarbonate buffer and digested with trypsin (0.01 μg of trypsin per 1 μg of protein) for 14 hr at 37°C. After trypsin digestion, the reaction was stopped by addition of formic acid to a final concentration of 5%. The obtained tryptic fragments were desalted by Discovery DSC-18 50 mg microcolumns (Sigma, USA), dried in vacuum, and re-dissolved in 3% ACN with a 0.1% FA solution prior to the LC-MS/MS analysis.

**LC-MS/MS Analysis**

The LC-MS/MS Analysis was performed on a TripleTOF 5600+ mass-spectrometer with a NanoSpray III ion source (ABSciex) coupled with a NanoLC Ultra 2D+ nano-HPLC system (Eksigent, USA). The HPLC system was configured in a trap-elute mode. For sample-loading buffer and buffer A, a mixture of 98.9% water, 1% methanol, and 0.1% formic acid (v/v) was used. Buffer B was 99.9% acetonitrile and 0.1% formic acid (v/v). The samples were loaded on a Chrom XP C18 trap 3 μm 120 Å 350 μm*0.5 mm column (Eksigent, USA) at a flow rate of 3 μl/min for 10 min and eluted through a 3C18-CL-120 separation column (3 μm, 120 Å, 75 μm*150 mm; Eksigent) at a flow rate of 300 nl/min. The gradient ranged from 5 to 40% of buffer B in 90 min, followed by 10 min at 95% buffer B and 20 min re-equilibration with 5% of buffer B. The information-dependent mass-spectrometer experiment included 1 survey MS1 scan, followed by 50-dependent MS2 scans. The MS1 acquisition parameters were as follows: mass range for the MS2 analysis was 300– 1250 m/z, and signal accumulation time was 250 ms. Ions for the MS2 analysis were selected on the basis of intensity with a threshold of 200 cps and a charge state ranging from 2 to 5. The MS2 acquisition parameters were as follows: the resolution of quadrupole was set to UNIT (0.7 Da), the measurement mass range was 200–1800 m/z, and signal accumulation time was 50 ms for each parent ion. Collision-activated dissociation was performed with nitrogen gas with a collision energy ramping from 25 to 55 V within a signal accumulation time of 50 ms. Analyzed parent ions were sent to a dynamic exclusion list for 15 sec in order to generate an MS2 spectra at the chromatographic peak apex. A β-Galactosidase tryptic solution (20 fmol) was run with a 15-min gradient (5-25% of buffer B) every 2 samples and between sample sets to calibrate the mass-spectrometer and to control overall system performance, stability, and reproducibility.

**LC-MS/MS Data Analysis**

LC-MS/MS data were converted to .mgf peak lists with ProteinPilot (version 4.5). For this procedure, we ran ProteinPilot in an identification mode with the following parameters: Cys alkylation by iodoacetamide, trypsin digestion, TripleTOF 5600 instrument, and thorough ID search with detected protein threshold 95.0% against UniProt human Protein knowledgebase (version 2013_03, with 150600 entries). For thorough protein identification, the generated peak lists were searched with the MASCOT (version 2.2.07) and the X! Tandem (CYCLONE, 2013.2.01) search engine against UniProt human Protein knowledgebase (version 2013_03), with a concatenated reverse-decoy dataset (with 301200 entries altogether). Precursor and fragment mass tolerance were set at 20 ppm and 0.04 Da, respectively. Database searching parameters included the following: tryptic digestion with 1 possible miss cleavage, static modifications for carbamidomethyl (C), and dynamic/flexible modifications for oxidation (M). For X! Tandem, we also selected parameters that allowed a quick check for protein N-terminal residue acetylation, peptide N-terminal glutamine ammonia loss, or peptide N-terminal glutamic acid water loss. Result files were submitted to the Scaffold 4 software (version 4.0.7) for validation and a meta analysis. We used the LFDR scoring algorithm with standard experiment-wide protein grouping. For the evaluation of peptide and protein hits, a false discovery rate of 5% was selected for both. False positive identifications were based on reverse database analysis.

## RESULTS AND DISCUSSION

**Fig. 1:**
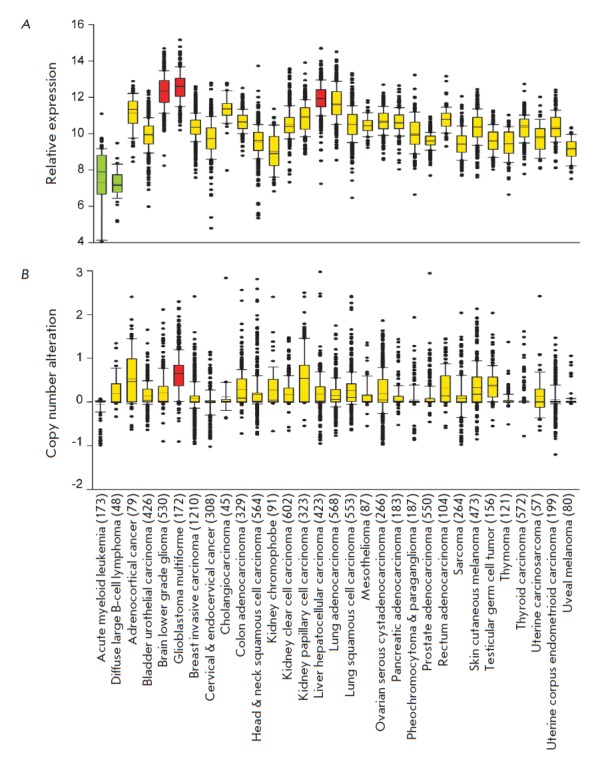
Relative expression level (*A*) and copy number variation (*B*) of the *PON2 *gene in tumors from patients with different types of cancer. Results were obtained by bioinformatic analysis of RNA and DNA sequencing data from the TCGA database. The number of patients for each cohort is indicated in brackets

In order to identify the types of malignancies for which PON2 can play a potentially important oncogenic role, we compared the expression levels of this protein using the RNA sequencing data from the TCGA (The Cancer Genome Atlas) database. Having analyzed the data obtained for more than 10,000 patients with 31 types of malignancies, we found that the highest expression level of PON2 is observed in liver and brain cancer (grade 1–3 gliomas and grade 4 glioblastoma), while the lowest expression level of this protein is typical of leukemia (myeloid leukemia and B-cell lymphoma) (*Fig. 1A*). In order to understand whether the observed disturbance of PON2 expression is caused by mutations in the respective gene, we analyzed the genomic DNA sequencing data to reveal any possible amplifications or deletions of this gene in different tumor types. One can see from *Fig. 1B* that amplification of the *PON2 *gene is typical of glioblastoma, while deletion of this gene is usually observed in leukemia. This result is in good agreement with our findings obtained by analyzing PON2 expression.

**Fig. 2:**
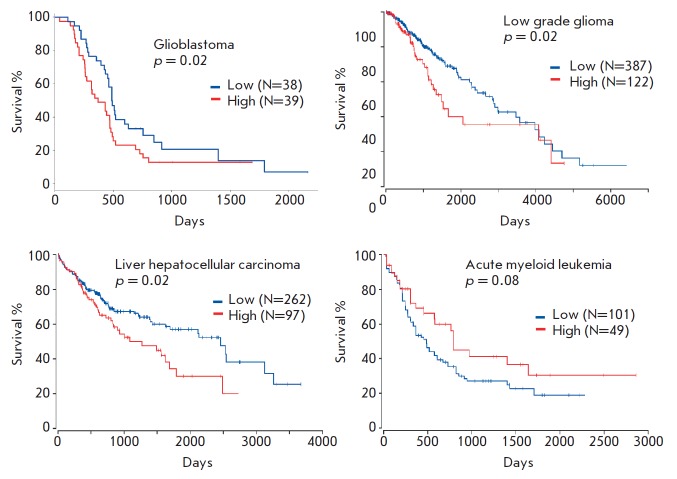
The Kaplan–Meier survival curve for patients with glioblastoma, low grade glioma, liver hepatocellular carcinoma and acute myeloid leukemia divided into two groups based on the PON2 expression level. Results were obtained by bioinformatic analysis of the TCGA database. The number of patients in each group and the *p *value (log-rank test) are indicated

In order to assess the effect of PON2 on the proliferation and resistance of tumor cells to therapy, we analyzed how the expression level of this protein is associated with the survival of patients with different types of cancer. The data in *Fig. 2* convincingly demonstrate that a high PON2 level correlates with poor prognosis for patient survival in liver cancer, glioma, and glioblastoma, while the opposite is true for leukemia: an elevated level of paraoxonase 2 is a good prognostic indicator. These results are fully consistent with our findings on the expression level and mutations in the *PON2 *gene.

The large necrotic zone emerging in the tumor center, its volume often being many times larger than the amount of viable tumor tissue, is a distinctive clinical feature of brain cancer [12]. Such a high level of cancer cell death is associated with an insufficient blood supply to glioblastoma and extremely limited space for growth. For this reason, glioblastoma cells are continuously exposed to the stress caused by the lack of nutrients and the toxic components released by neighboring dying cells. A similar situation is observed in the liver, since potentially harmful substances are delivered from blood to this organ. Hence, it is fair to assume that PON2 plays a crucial role in liver and brain cancer cells, as it helps them adapt to existence in an environment with a high concentration of toxic metabolic products and lack of nutrients. Therefore, selection of tumors with an increased PON2 expression level may take place as these tumors develop and one of the reasons is the amplification of the respective gene. Contrariwise, leukemia cells exist in a favorable environment that is rich in oxygen and nutrients and contains no potentially toxic substances. As a result, they do not require a high PON2 expression level; conversely, the reduced PON2 level seems to be responsible for the more aggressive phenotype of these tumor cells.

**Fig. 3:**
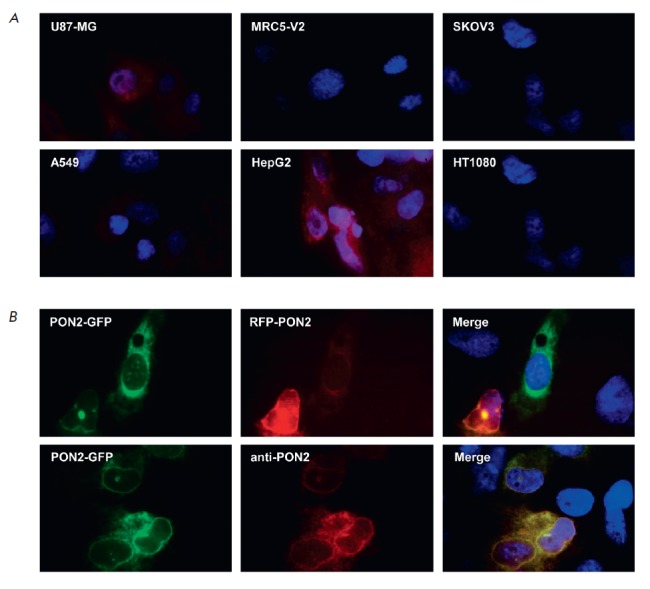
*A *– Immunofluorescent staining of different cell lines with anti-PON2 antibodies. *B *– Fluorescence images of cells cotransfected with the pTagRFP-C-PON2 and pTurboGFP-N-PON2 plasmids (upper panel) and cells transfected with the pTurboGFP-N-PON2 plasmid and then stained with anti-PON2 antibodies (lower panel)

In order to glean more information about the functions of PON2 in tumor cells, we stained 6 cell lines (U87-MG – glioblastoma; MRC5-V2 – embryonic lung; SKOV3 – ovarian carcinoma; A549 – lung carcinoma; HepG2 – liver carcinoma; and HT1080 – fibrosarcoma) with antibodies specific to this protein. Our results demonstrated that staining with the highest intensity is observed in glioblastoma and liver carcinoma cells, which is consistent with the bioinformatic analysis data (*Fig. 3A*). In all the analyzed cell types, PON2 was localized in the perinuclear region. Since the quality of immunocytofluorescence staining did not allow us to accurately identify the localization of PON2 in cells, the next step was to study the localization of exogenously expressed paraoxonase 2. For this purpose, we cotransfected U87-MG cells with plasmids pTagRFP-C-PON2 (encodes the red fluorescent protein linked to the N-terminus of PON2) and pTurboGFP-N-PON2 (encodes the green fluorescent protein linked to the C-terminus of PON2) and stained the transfected cells with anti- PON2 antibodies. One can see in *Fig. 3B* that PON2 was predominantly localized around the nucleus, regardless of where the fluorescent protein was inserted.

**Fig. 4:**
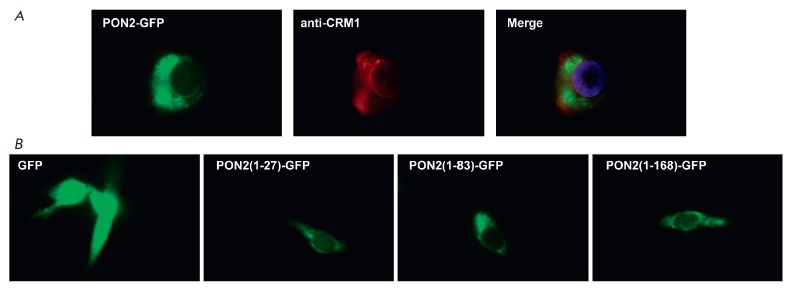
*A *– Fluorescence images of cells transfected with the pTurboGFP-N-PON2 plasmid and then stained with anti-CRM1 antibodies. *B *– Fluorescence images of cells transfected with plasmids encoding different fragments of PON2 (1–27 a.a.; 1–83 a.a.; 1–168 a.a.) or GFP alone as a control

In order to determine PON2 localization more accurately, we stained the transfected cells with antibodies specific to the CRM1 protein, a marker of the nuclear envelope. One can see in *Fig. 4A* that PON2 around the nucleus is completely colocalized with CRM1, suggesting that a significant portion of PON2 in the cell resides on the nuclear envelope.

Next, we attempted to identify the amino acid sequence of PON2 required to ensure localization of this protein on the nuclear envelope. With this in mind, we created plasmids encoding three PON2 fragments (1–27 a.a.; 1–83 a.a.; and 1–168 a.a.) carrying the green fluorescent protein at their N-terminus. *Figure 4B* demonstrates that the first 27 amino acids of PON2 encoding the transmembrane segment of this protein are sufficient for ensuring localization of PON2 on the nuclear envelope.

Finally, we employed the LC-MS/MS method to identify the intracellular proteins interacting with PON2. We transfected the cells with a plasmid encoding either PON2 or the control protein, labeled with the Halo Tag at its C-terminus. Magnetic particles with Halo Tag ligand were used to isolate exogenous PON2 and the proteins interacting with it. Subsequent LC-MS/MS analysis allowed us to identify 286 proteins coprecipitating with PON2. Among those, 168 proteins were also detected in the control sample, while 119 proteins interacted exclusively with PON2, rather than with the control protein (*Table*). Among the proteins exhibiting a unique interaction with PON2, there were six localized on the nuclear envelope (CACYBP, TMPO, S100A6, RAN, UBXN4, and TOR1AIP1). It is important to mention that the highest number of peptides (not counting PON2) was identified for the CACYBP protein, which can be indicative of a high intensity of interaction between CACYBP and PON2.

## CONCLUSIONS

A large body of studies describing the functions of PON2 in several types of malignancies has been published over the past year. In order to acquire more general information about the role played by this protein in various types of malignancies, we have, for the first time, analyzed the expression level and mutations in the *PON2 *gene in 31 types of malignancies and investigated the association between the expression level of PON2 and patient survival. Our findings demonstrate that the highest level of PON2 expression is observed in solid tumors, in particular in brain tumor and liver cancer. Amplification of the *PON2 *gene and correlation of its expression with unfavorable prognosis of survival are also typical of these tumors. Contrariwise, hematologic malignancies are characterized by a low level of this protein, deletions of the respective gene, and correlation of the level of PON2 expression with a favorable prognosis. It is known that PON2 plays various functions in the cell, such as lactone cleavage, reduction of free radical production in mitochondria, and protection of membrane lipids against peroxidation. According to the data on the localization of this protein in the cell and on its interaction with other proteins, it is fair to assume that PON2 in tumor cells mainly protects the intracellular membranes against oxidation and, possibly, prevents free radicals from percolating through the nuclear envelope and damaging the genetic material contained in the cells. However, further research is needed for definitive confirmation of this hypothesis.

**Table:** List of the proteins coprecipitated with PON2

Gene	MW	N	Gene	MW	N	Gene	MW	N	Gene	MW	N
PON2	39	9	RPS20	13	2	RPS18	18	1	SNU13	14	1
CACYBP	26	7	RPS7	22	2	RPL12	18	1	HNRNPD	31	1
RPS10	19	6	RPL11	20	2	RPL14	23	1	FKBP3	25	1
RPS19	16	6	RPL30	13	2	ADD3	74	1	PTRF	43	1
HSPA1A	70	6	NARS	63	2	AIDA	35	1	DHX15	91	1
PARP1	113	6	DTD1	23	2	CTNNBL1	65	1	PSME3	30	1
RPS5	23	5	EIF2S2	38	2	FLJ51636	12	1	DEK	43	1
EIF3A	75	5	EIF3G	36	2	CCDC124	26	1	DR1	19	1
EIF5	49	5	EXOSC2	33	2	COL12A1	333	1	S100A11	12	1
MANF	21	5	FARSLA	58	2	CSTB	11	1	S100A6	10	1
NELFE	43	5	GTF2F2	28	2	DCD	11	1	SEC61G	8	1
AHNAK	629	5	HDGF	27	2	MCM4	97	1	PTD004	20	1
FLJ20643	32	5	CDC37	44	2	DNAJC17	35	1	SARNP	24	1
ERP29	29	4	HYPK	15	2	DNAH10	515	1	SRSF1	28	1
EIF3J	29	4	IMPDH2	56	2	EEF1B2	25	1	SRSF3	19	1
EIF4B	69	4	TIMM8B	9	2	GTF2F1	58	1	STK10	112	1
FEN1	43	4	PYM1	23	2	GAPDHS	45	1	STK24	23	1
METAP1	43	4	CWC27	54	2	RAN	24	1	SARS	59	1
RPL9	22	3	PABPC1	71	2	HNRNPUL1	96	1	SNRPF	10	1
ATP5O	23	3	PTBP1	57	2	HIST1H2AB	14	1	CWC15	27	1
DNAJB1	38	3	PAWR	37	2	HIST1H4A	11	1	STMN1	17	1
HNRNPA1	39	3	PDIA3	57	2	IGF2BP1	63	1	TOR1AIP1	66	1
KRT2	65	3	MAGOHB	17	2	ITIH3	100	1	TCEAL4	25	1
TMPO	75	3	PBDC1	26	2	LIMS1	38	1	TUBA1C	50	1
TIMM8A	11	3	SNRPB	25	2	ZFYVE28	96	1	PTPN1	50	1
PA2G4	45	3	TARS	83	2	MESDC2	26	1	YARS	59	1
FAM50A	40	3	NSUN2	86	2	METAP2	53	1	UBXN1	33	1
SKIV2L2	118	3	MRPS11	21	1	MAPRE1	30	1	UBXN4	57	1
TCEA1	34	3	RPS16	16	1	DNAJC19	12	1	HDLBP	141	1
RPS15	17	2	RPS17	16	1	NAA50	19	1			

Note. Gene name (Gene), molecular weight in kDa (MW), and the number of unique peptides identified by LC-MS/MS mass spectrometry (N) are indicated.

## Abbreviations

PON2: paraoxonase 2
TCGA: The Cancer Genome Atlas
